# Depression and Risk of Unintentional Injury in Rural Communities—A Longitudinal Analysis of the Australian Rural Mental Health Study

**DOI:** 10.3390/ijerph14091080

**Published:** 2017-09-18

**Authors:** Kerry J. Inder, Elizabeth G. Holliday, Tonelle E. Handley, Lyn J. Fragar, Tony Lower, Angela Booth, Terry J. Lewin, Brian J. Kelly

**Affiliations:** 1 School of Nursing and Midwifery, University of Newcastle, Callaghan, NSW 2308, Australia; kerry.inder@newcastle.edu.au; 2 Hunter Medical Research Institute, New Lambton, NSW 2305, Australia; elizabeth.holliday@hmri.org.au; 3 Centre for Brain and Mental Health Research, School of Medicine and Public Health, University of Newcastle, Callaghan, NSW 2308, Australia; angela.booth@newcastle.edu.au (A.B.); terry.lewin@hnehealth.nsw.gov.au (T.J.L.); brian.kelly@newcastle.edu.au (B.J.K.); 4 School of Public Health, University of Sydney, Sydney, NSW 2006, Australia; lyn.fragar@sydney.edu.au (L.J.F.); tony.lower@sydney.edu.au (T.L.); 5 Hunter New England Mental Health, Newcastle, NSW 2300, Australia

**Keywords:** injury, depression, affective disorder, rural mental health, risk factors, longitudinal analysis

## Abstract

Limited longitudinal research has examined relationships between depression and injury, particularly in rural contexts. This paper reports cross-sectional and longitudinal analyses from the Australian Rural Mental Health Study (ARMHS) exploring relationships between “probable depression” episodes and unintentional injury. Participants completed four surveys over five years. Multivariate logistic regressions were employed to assess the causal effect of prior depression episodes on subsequent injury risk. Of 2621 baseline participants, 23.3% experienced a probable depression episode recently and 15.9% reported a serious injury during the previous 12 months. Factors associated with a 12-month injury at baseline included male gender, being unemployed or unable to work, being involved in a serious incident, hazardous alcohol use, and having experienced a recent depression episode. Longitudinal analyses revealed that probable depression was significantly associated with subsequent unintentional injury (OR 1.68, 99%CI 1.20–2.35), as was male gender (OR 1.39, 99%CI 1.06–1.82), while alcohol consumption did not mediate these relationships. Campaigns to reduce the impact of mental illness should consider unintentional injuries as a contributor, while injury prevention initiatives may benefit from addressing mental health issues. Such strategies are particularly important in rural and remote areas where injuries are more common and mental health services are less readily available.

## 1. Introduction

Injuries represent a substantial public health issue worldwide. Acute injury is a major cause of hospitalisations in Australia and is in the top five burdensome disease groups, accounting for 8.8% of total Disability-Adjusted Life Years [1]. In 2012–2013 there were 447,000 injuries requiring hospitalisation in Australia, accounting for 6% of all hospitalisations, with a further 1.8 million emergency room presentations [2]. The costs of injuries are significant and range from financial to social, human, and organisational. Depending on its severity, unintentional injury may lead to temporary or permanent loss of income, as well as costs associated with initial treatment and ongoing care and rehabilitation [3]. It may also impact on the individual’s ability to work; their emotional wellbeing; and relationships with family, friends, and the community [4].

Some sections of the population have been identified as being more at risk of injury than others [5]. For example, hospitalisations are more common among males than females, and more common among older people than younger people [2]. In addition, the rate of hospitalised injury for rural residents is 1.5 times higher than that of urban residents [5], increasing with the degree of remoteness from 1.3 times for outer regional residents to 1.8 times for remote residents and 2.2 times for very remote residents [2]. In light of such findings, targeted regional- or community-level programs have been advocated, specifically focused on reducing accidents, injury, poisoning, and suicide [6]. Rural and remote Australians have also been identified as a group that could benefit from specific injury-prevention initiatives, as outlined in the “National Injury Prevention and Safety Promotion Plan: 2004–2014” [7]. The reasons for increased injuries in rural and remote areas are various; many of these factors are occupational and include a higher proportion of occupations involving the use of machinery, working in isolation [8], and long working hours [9]. Other factors include more road travel and roads which are often of poorer quality [8], poorer compliance with safety regulations, and greater risk-taking behaviours [9].

Mental health issues following injury have been widely reported, and may range from general anxiety and distress to psychiatric diagnoses such as Post-Traumatic Stress Disorder. More recently, research has suggested that the relationship between unintentional injury and mental health issues may be bi-directional, with mental illness acting as a predisposing factor for future injury. For example, an American study found that 20% of patients admitted to hospital for an injury had a diagnosable mental illness; these individuals had twice the risk of an initial injury and 4.5 times the odds of injury recidivism compared with patients who did not have a mental illness [10]. Likewise, a population-based study in Finland found that depressive symptoms increased the odds of unintentional injury by 52% [11]. These cross-sectional findings have been supported by some longitudinal research; for example, a study by Cameron et al. [12] using health service data reported that individuals hospitalised for unintentional injury were more likely to experience mental health concerns in the ten years prior to the injury’s occurrence. A similar data linkage study in Australia found that males were more likely to experience an injury-related hospitalisation if they had previously been engaged with mental health services, particularly if their mental health care had been interrupted [13]. Longitudinal relationships between occupational injury and depression have also been examined [14], with gender differences detected in the patterns of association: males experiencing workplace injury were found to be more vulnerable to post-injury depression (compared with non-injured males), while female workers with depression were more likely to be injured at work (compared with non-depressed female workers).

Multiple symptoms of depression, including difficulty concentrating, insomnia/fatigue, and psychomotor retardation or agitation, are potential mechanisms through which depression may increase the likelihood of unintentional physical injury. These symptoms may contribute to decreased reaction times and impaired motor skills [15], which in turn contribute to risk of injury. Factors such as fatigue are often the focus of public health and safety campaigns, such as road safety and workplace safety approaches, as a means of accident prevention. However, little research attention has been given to depression as an underlying psychosocial cause of these symptoms. It is also possible that alcohol may play a pivotal role in these associations, as alcohol consumption is frequently associated with unintentional injuries, and is also often used as a means of self-medication by those experiencing mental illness [16]. Considering that alcohol consumption is more common in rural than urban areas [17], and that these areas also experience higher rates of unintentional injury, exploring the potential mediating role that alcohol consumption may play between depression and injury in rural areas is important. Research exploring these concepts is primarily cross-sectional and more longitudinal research would be beneficial [18]. Similarly, research focusing specifically on rural populations is lacking.

We acknowledge that “rural” definitions vary widely and that the specific elements of “rurality” that impact on day-to-day functioning and health outcomes may be difficult to ascertain and apportion [19,20,21]. For present purposes, “rural” is conceptualised as a simple proxy term for the set of geographically dispersed health determinants that prevail in non-metropolitan areas [22], including: socio-economic disadvantage (greater in many rural areas); greater exposure and vulnerability to environmental adversity (e.g., severe drought) and its socio-economic sequelae; vulnerability to change and related impacts on community infrastructure (e.g., out-migration for education or employment); poorer access to health and social services; and geographic isolation (and, more specifically, social isolation) [21,22]. However, in reality, an array of regional and contextual factors probably also need to be taken into account, including positive factors such as community resilience and attachment [19,23].

The Australian Rural Mental Health Study (ARMHS), which utilises a population-based longitudinal cohort of adult men and women, was designed by our research team to investigate the determinants and outcomes of common mental disorders in rural and remote communities [24]. With respect to environmental and contextual factors, ARMHS-based analyses to-date have tended to focus on broad psychosocial indices, such as remoteness and accessibility of services [22], social support [25], and financial hardship [26], or on subsets of individuals with particular experiences, such as exposure to prolonged drought [27] or traumatic events [28]. One previous analysis from ARMHS replicated the findings of many urban-based studies, reporting that prior depression was significantly associated with unintentional injury in a domestic or public setting, while unintentional injury in a high-risk setting (such as the workplace or a sporting activity) was significantly associated with current psychological distress [29]. These findings persisted after controlling for factors such as farm residence and level of remoteness. Other factors that contributed to unintentional injury included older age, being unemployed or unable to work, and male gender. A limitation of this analysis was its cross-sectional nature, meaning that it was unable to explore the temporal relationship between injury and depression, and was reliant on participant recall to determine the order of occurrence of injuries and mental health concerns.

The current paper builds on the previous cross-sectional report by Fragar et al. [29] via an examination of the temporal relationship between depression episodes and risk of subsequent non-fatal unintentional injury in rural communities in Australia using longitudinal data from ARMHS. Secondly, it explores whether this relationship is mediated by alcohol use.

## 2. Materials and Methods

### 2.1. Participants

The ARMHS cohort [22,24] was recruited from rural communities using a household sampling framework based on the Australian Bureau of Statistics (ABS) geographic classification, specifically the Accessibility/Remoteness Index of Australia Plus (ARIA+), with over-sampling of remote and very remote regions of New South Wales (NSW). The ARIA+ is a continuous index score ranging from 0 to 15, where higher scores indicate greater remoteness based on the average estimated road distance between a location and the nearest service centre. Five geographical categories are typically identified: metropolitan (average ARIA+ of 0 to 0.20, with populations >100,000); inner regional (0.21 to 2.40); outer regional (2.41 to 5.92); remote (5.93 to 10.53); and very remote (>10.53). The ARMHS sample excluded metropolitan centres and comprised household residents aged 18 years or older living in private dwellings, drawing from an area covering approx. 70% of non-metropolitan NSW [22].

Participants gave written consent to complete each survey. Baseline postal surveys were completed between 2007 and 2008, with follow-up postal surveys at 12 months, 3 years and 5 years after baseline. ARMHS was approved by the Human Research Ethics Committees of the University of Newcastle (reference: H-145-1105a) and the University of Sydney (reference: 13069), and the relevant health districts. The reporting of ARMHS findings accords with Strengthening the Reporting of Observational Studies in Epidemiology (STROBE) guidelines [30].

### 2.2. Measures

#### 2.2.1. Outcome and Exposure Variables

The primary outcome of unintentional injury was assessed by asking participants to report the most serious injury they had experienced in the last 12 months that required treatment from a doctor or hospital. Injury-related data were classified using the World Health Organisation’s International Classification of Diseases (ICD-10) [31] for type of injury and the World Health Organization’s International Classification of External Injury (ICECI) [32] to describe how the injury came about and where it occurred. Each case was reviewed individually and some cases were re-classified as chronic disease rather than acute injury. Injury data were collected as part of each postal survey.

The likelihood of having experienced a recent depression episode (i.e., during the designated study wave) was derived using a combination of three survey scales and questions. For each measure, an indicator (0/1) of possible depression was generated using the following thresholds:

- Kessler Psychological Distress Scale (K10) [33] (total score > 15). The K10 assesses the frequency of ten relatively non-specific symptoms over the past four weeks on a five-point scale ranging from “None of the time” to “All of the time”. Symptoms include feeling depressed, tired, nervous, or hopeless.
- Patient-Health Questionnaire-9 (PHQ-9) [34] (total score ≥ 5). The PHQ-9 is a depression severity measure which assesses the frequency of nine symptoms of depression over the previous two weeks on a four-point scale from “Not at all” to “Nearly every day”. Symptoms include having little interest or pleasure in doing things, feeling down, depressed or hopeless, and feeling tired or having little energy.
- A survey question worded: “Has a doctor ever told you that you have depression, stress or anxiety” (yes). Responses to this item were used in a cumulative manner across study waves (i.e., scored as positive from the wave at which it was first endorsed and thereafter).

The sum of depression indicators across these three items was then used to classify the likelihood of having experienced a recent depression episode during that wave, as follows: 0 = Unlikely; 1 = Somewhat likely; ≥2 = Probable depression episode. The rationale for this approach was that there needed to be multiple lines of evidence to categorise an episode as “probable”, either in the form of several self-reported depression symptoms that were also causing distress (i.e., positive on both standardised measures), or threshold-level symptoms within the context of acknowledged external clinical confirmation (i.e., positive on one standardised measure plus “ever told by doctor”). The basic logic underlying the derivation of this depression likelihood index was akin to that often used in structured diagnostic assessments, where point prevalence estimates are based on a combination of lifetime disorder assessments and concurrent but less detailed information about recent symptoms [35].

The accuracy of this derived depression variable was assessed using a subset of participants for whom a diagnostic interview (the World Mental Health Composite International Diagnostic Interview; WMH-CIDI [36]) had been completed at baseline. Accuracy was assessed by fitting a logistic regression model with ordinal depression likelihood as the predictor and International Classification of Diseases (ICD-10) lifetime affective disorder as the outcome. Diagnostic accuracy was assessed by constructing a Receiver Operator Characteristic (ROC) Curve, calculating the Area Under the Curve (AUC), and calculating the sensitivity, specificity, positive likelihood ratio, and negative likelihood ratio for the various depression categories/cut-points.

#### 2.2.2. Potential Confounders

The potential confounders for which adjustment was required in multivariate models were identified by constructing a directed acyclic graph (DAG) describing the presumed causal relationships among probable depression, injury, and a range of potential confounders/mediators. Based on the DAG (shown in Supplementary Figure S1), the following variables were considered potential confounders and included as adjustments in the multivariate regression: age; gender; education; marital status; employment status; chronic disease (heart attack or angina, other heart disease, high blood pressure, stroke, cancer, or diabetes); and unintentional injury in the last 12 months (i.e., the outcome measure at the previous timepoint).

Alcohol consumption was viewed as a likely contributor to the causal pathway between depression and injury, as alcohol use has been linked to both depression [37] and injury [38] independently. Thus, adjustment for alcohol consumption was not desired when estimating the total causal effect of probable depression episodes on subsequent injury, and hence this was not included in the model. A secondary question, however, was estimation of the indirect effect of depression on subsequent injury mediated through alcohol consumption. The 10-item World Health Organization’s Alcohol Use Disorders Identification Test (AUDIT) [39] was used to assess alcohol use during the past 6 months (low risk, 0–7; hazardous use, ≥8).

### 2.3. Statistical Analysis

All statistical analyses were programmed using SAS v9.4 (SAS Institute, Cary, NC, USA). Group differences were tested using ANOVA for continuous variables and chi-square tests for categorical variables.

#### 2.3.1. Estimating the Total Causal Effect of Probable Depression on Injury Risk

To assess the total causal effect of prior depression on subsequent injury risk, multivariate logistic regressions using generalized estimating equations (GEE) were employed to account for potential correlations between repeated measures within individuals, and between individuals within a household. An exchangeable correlation structure was assumed.

To ensure appropriate temporal ordering between the exposure and outcome, a first-order lagged exposure (likelihood of depression) was used. That is, injury outcomes from Surveys 2–4 were linked to the depression likelihoods measured at Surveys 1–3, respectively. Covariates were similarly lagged, to ensure that potential confounders were ancestors (i.e., causes of), rather than descendants of (i.e., caused by) the outcome. We did not adjust for the lagged outcome, since there was little evidence of autocorrelation of injury within individuals over time. Parameter estimates are presented as Odds Ratios with 99% Confidence Intervals. Associations were considered statistically significant at *p* < 0.01.

#### 2.3.2. Handling of Missing Data: Multiple Imputation

The primary analysis was a complete case analysis, assuming data were missing completely at random. For each participant, this analysis included any non-missing outcome from Surveys 2, 3 and 4 that also had non-missing exposure and covariate data from the prior survey. The total number of participants with baseline data was 2639. Consequently, the maximum possible number of exposure-outcome combinations was 3 per participant (3 × 2639 = 7917).

In assessing missing data, we distinguished between exposure-outcome combinations missing due to study dropout (no survey returned for the exposure and/or the outcome: whole-wave missingness) and item nonresponse (both surveys returned, but with some variables missing: within-wave missingness). A total 3998 exposure-outcome combinations had whole-wave missingness for the exposure and/or the outcome. We did not consider data imputation in these cases, since there was no participant information available to inform imputed values for the missing wave. Of the remaining 3919 exposure-outcome combinations, 250 (~6%) had within-wave missingness (missing one or more of exposure, covariates, or outcome). For these, multiple imputation was performed assuming data were missing at random, using available data from the affected wave(s), as well as other available waves for the participant.

Multiple imputation (*N* = 25 sets) was performed using a fully conditional specification as implemented in the MI procedure within SAS. The imputation model included all variables used in the causal regression model, plus additional variables predicting missingness, and/or observed values of missing variables. GEE models were then fitted to the individual imputed datasets and parameter estimates were combined using the SAS MIANALYZE procedure.

#### 2.3.3. Estimating the Indirect Effect of Probable Depression Mediated through Alcohol Consumption

After finding evidence for a total causal effect of probable depression episodes on subsequent injury risk, a secondary question was whether a meaningful component of this effect was mediated via alcohol consumption. The indirect effect of probable depression on injury mediated through alcohol was assessed using the regression-based approach to mediation proposed by Baron and Kenny [40]. This involves fitting a sequence of four regression models:

1. Logistic regression to estimate the total effect of probable depression on injury (primary analysis);
2. Logistic regression to estimate the effect of probable depression on alcohol consumption;
3. Logistic regression to estimate the effect of alcohol consumption on injury; and,
4. If all effects in Steps 1–3 are significant, logistic regression to estimate the effect of alcohol consumption on injury, adjusted for probable depression.

Given that all outcomes were binary, each model was fitted using the same multivariate logistic regression (GEE) framework as the primary analysis, adjusting for lags of all model covariates. In each case, a lagged exposure was also used. Reported analyses were complete case, since causal modelling provided similar results for complete case and multiply imputed data.

## 3. Results

### 3.1. Sample Characteristics

Recruitment to ARMHS is described in detail elsewhere [22,24]. The initial survey was completed by 2639 individuals (from 1879 households), with an under-representation of younger people (i.e., lower contact and survey completion rates) [24]; in other respects, the sample was consistent with regional profiles, taking into account the over-sampling from remote/very-remote regions. Selected characteristics of the ARMHS sample at baseline are reported in Supplementary Table S1 (e.g., average age: 55.6 ± 14.7 years; 59.4% female; 75.2% married/de facto relationship; 24.9% farm residence; and 26.1% from remote/very remote regions). A full description of participation through the four waves of ARMHS is shown in Supplementary Figure S2. Participants lost to follow-up were generally younger at baseline (52.4 ± 16.0 years) than those who were retained (56.3 ± 13.9 years), F_(1, 2619)_ = 40.27, *p* < 0.001; they also had lower levels of education (School Certificate only or lower: 35.4% vs. 28.0%, χ^2^_(2)_ = 13.45, *p* < 0.001) and a higher tendency to be unemployed or permanently unable to work (9.6% vs. 7.0%, χ^2^_(2)_ = 9.87, *p* = 0.007). There were no differences in retention status across other baseline socio-demographic or health characteristics, such as gender, marital status, or recent depression (see Supplementary Table S1).

Of 2621 participants at baseline with relevant indicators, over one-fifth (*N* = 611; 23.3%) were identified as having experienced a “probable depression” episode recently and over half (*N* = 1431; 54.6%) as having been “unlikely” to do so. A smaller proportion (*N* = 419; 15.9%) reported experiencing a serious injury requiring hospitalisation or medical treatment during the previous 12 months. The most common injury was a strain or sprain, followed by a cut, laceration or abrasion, or a fracture. Injuries most commonly occurred in the home, followed by a sport or recreation activity, or on a farm, and were typically the result of a fall, trip or slip, body stressing, or being hit by a moving object.

Overall, the derived depression likelihood variable showed moderate accuracy for predicting individuals with an ICD-10 lifetime affective disorder (AUC = 0.837; 99%CI 0.788–0.886). A cut-point of “probable depression” (i.e., positive on 2 or more indicators) provided modest sensitivity (65.7%) but relatively high specificity (81.4%), supporting its use in the current analyses (see Supplementary Table S2 for further details). However, importantly, this index was designed to identify individuals who were more likely to have experienced a depressive episode during the designated study wave. Across the four study waves, 1457 surveys were categorized as indicating a recent “probable depression” episode, amongst which 42.3% were positive on all three indicators, 31.3% were positive on the “ever told by doctor” indicator plus one other, and a further 26.4% were positive on both symptom measures (K10, PHQ-9) (see Supplementary Table S3 for details by study wave).

The characteristics associated with recent depression likelihood at baseline are shown in Table 1. Those reporting a higher likelihood (i.e., probable or somewhat likely) were younger than those without a depression episode, and were proportionately more likely to be female, not partnered, and unemployed or unable to work. They were also proportionately more likely to have been involved in a serious accident in the past 12 months and to report hazardous alcohol use in the past 6 months. Factors associated with a 12-month injury at baseline included male gender, being unemployed or unable to work, being involved in a serious accident, and hazardous alcohol use. In addition, there was a strong association between increasing depression likelihood and unintentional injury.

### 3.2. Primary Analysis

As shown in Table 2, at each of Surveys 2–4, there was evidence of a linear relationship between injury frequencies and increasing likelihood of earlier depression. We note that the rate of recurrent injury was relatively low. The 585 total injuries recorded across Surveys 2–4 were reported by 481 unique participants. Of these, 389 reported a single injury, 80 reported injuries at two surveys and 12 reported injury at all three follow-up surveys.

The longitudinal analysis showed that, on a univariate basis, unintentional injury was more common among those with a prior probable depression episode, as well as among males and those who were not partnered (see Table 3). Results from the primary multivariate logistic model (complete case analysis) were largely consistent with this. Effect estimates were similar to those from univariate analyses, suggesting little confounding of the univariate effect by measured confounders. Probable depression was significantly associated with unintentional injury, as was male gender. The interaction between time lapse and depression likelihood was non-significant (*p* = 0.680); thus, the time lapse interaction terms were not retained in the model. This suggests that the estimated effect of depression on subsequent injury risk was not substantially moderated by time lapse between surveys.

In the multivariate analyses using multiply imputed data, results (not shown) were similar to those from complete case analyses. In particular, the estimated effects of depression likelihood were almost identical, with probable depression episodes associated with a 1.69-fold increase in the odds of subsequent unintentional injury (99%CI 1.31–2.18, *p* < 0.001). No other variable was associated with injury at *p* < 0.01 in this model (male gender, *p* = 0.023; not partnered, *p* = 0.053).

### 3.3. Mediation Analysis

Given the significant association between depression and subsequent injury, further regressions were performed to determine whether part of this effect was mediated by alcohol use. The mediation analysis was performed in four steps (see Methods). Step 1 simply reproduced the adjusted total causal estimate of depression likelihood on subsequent injury risk as shown in Table 3.

In Step 2, current alcohol consumption was regressed on depression likelihood at the previous wave, showing that participants with probable depression episodes had a 1.6-fold increase in the likelihood of subsequent high-risk alcohol use (OR 1.55, 99%CI 1.09–2.21). In Step 3, injury was regressed on alcohol use at the previous wave (OR 1.11, 99%CI 0.76–1.63, *p* = 0.474). No association was observed between past alcohol use and unintentional injury; therefore, Step 4 was not indicated. Taken together, these results suggest that the effect of depression on subsequent injury risk was not mediated by alcohol consumption.

## 4. Discussion

The current findings build on those by Fragar et al. [29] by contributing a longitudinal analysis to supplement previous cross-sectional findings. Similarly to Fragar et al. [29], the current longitudinal analysis found a significant relationship between 12-month unintentional injury and prior depression, providing stronger evidence for a temporal relationship between these factors. Whereas the cross-sectional study relied on participant recall to establish the order of onset of depression and injuries, the current analysis measured each variable across four timepoints to more accurately detect new occurrences across the study period.

These findings point to the importance of mental health support, a particularly salient issue in rural and remote Australia, where service availability is often poor. In addition to the psychological effects of mental illness, depression has been linked to a range of other vulnerabilities including chronic diseases such as heart disease, stroke, and diabetes mellitus [41]. Our findings support previous evidence from urban populations which suggests that physical injuries may be another vulnerability for those experiencing mental illness.

The present analysis also replicated the findings of Fragar et al. of a gender effect, with males being 39% more likely to report an injury over the course of the study. This is largely consistent with previous research, including an analysis of hospital admissions data by the Australian Institute of Health and Welfare showing that across Australia males were 30% more likely than females to be hospitalised due to injury [2].

In addition to the similarities between the present study and the cross-sectional analysis by Fragar et al. [29], there were also some differences between the findings. While Fragar et al. [29] reported a significant relationship between injury and current alcohol use (which is reflected in the Table 1 findings), alcohol was not found to be a significant mediator between depression and subsequent injury in the present analysis. While this does not indicate that there is no relationship between alcohol and depression, or between alcohol and injury, it does suggest that alcohol use is not the mechanism through which the link between depression and injury occurs. That is, the effects of depression on the likelihood of injury were independent of alcohol use, and strategies to improve injury prevention may be informed by this. The earlier cross-sectional analysis [29] also reported significant relationships between both unemployment and living on a farm and injury, which were only partially replicated in the present analyses. Considering that the analysis by Fragar et al. [29] was cross-sectional, while the present analysis was time-lagged to predict next-wave injury, it is possible that the previously identified factors do not act as longer-term predictors of future injury.

There are many potential implications of the current findings. From an intervention perspective, they suggest that injury prevention initiatives may benefit from considering mental health issues as a target, as poor mental health both prior to and following an injury may have considerable consequences. Alternatively, given some evidence of a bi-directional relationship between these constructs, it may also be beneficial to consider unintentional injuries as a factor in campaigns to lessen the impact of mental illness. From a public health perspective, these results are applicable in many settings. Injury prevention is a key priority for many workplace health and safety policies, and recognising the relationship between depression and injury in these settings may contribute to the reduction of workplace injuries [14]. For example, the “Psychological health and safety in the workplace” document released by the Standards Council of Canada [42] recognises that workplaces with a positive approach to psychological health tend to experience lower injury rates; the current results support this and suggest that positive approaches to mental health in the workplace may be applied internationally as a means of injury prevention.

Considering that the mean age of the ARMHS sample at baseline was 55.6 years (SD 14.7), our findings may also have implications for injury prevention among older adults. A Finnish study reported a link between depressive symptoms and unintentional injury among 45–74 year olds after controlling for age, gender, and a range of physical measures [11]. Likewise, a systematic review and meta-analysis of the relationship between depressive symptoms and falls among older adults found that these factors were consistently related, despite the use of different measures and varying follow-up lengths between studies [43]. While the authors acknowledge that the mechanism driving this relationship is currently unknown, they recommend that screening older people in falls prevention programs could identify those at greater risk, and that targeted interventions may be warranted for these individuals. A World Health Organisation study exploring risk factors for falls among older people in low- and middle-income countries also reported that depression was a significant contributor, indicating that these findings apply across both high-income and developing countries [44]. Further research exploring the mechanisms driving this relationship would be greatly beneficial to inform injury prevention approaches for this age group. In particular, it is possible that certain symptoms of depression, such as those associated with melancholia (e.g., psychomotor disturbance, interrupted sleep, impaired concentration and working memory [45]) may be more strongly related to injury than symptoms associated with low mood, and further research investigating sub-types of depression may provide more clarity around this.

Rural and remote residents were identified as one of eight key target groups under the “National Injury Prevention and Safety Promotion Plan: 2004–2014” [7]. Priority activities outlined under this plan included more accurate and detailed data collection around rural injuries, raising awareness among community members about the impact of various injuries on rural and remote communities, and advocating for greater attention and resources to address injuries in rural and remote areas. This document also recognised the challenges associated with implementing injury prevention initiatives in areas where relatively small populations are spread throughout large geographical areas, and acknowledged that strategies that are successful in major cities may not be applicable across rural regions. However, this plan is now outdated and has not been renewed, and as a result there is no current national injury prevention plan in Australia, despite unintentional injuries continuing to be a major cause of disability and mortality.

We acknowledge that the current study has several limitations. Firstly, and most notably, our outcome measure inquired about injuries that required treatment from a doctor or hospital. It is possible that the threshold for injuries that require treatment is higher for rural and remote residents than for urban residents, due to the increased difficulty in accessing treatment. That is, an injury may need to be more severe for a rural or remote resident to seek professional treatment. This may have affected our injury prevalence estimates, as well as the strength of the relationship between prior depression and injury in this study.

Secondly, our assessment of the likelihood of having experienced a recent episode of depression was based on a combination of three self-report measures collected at each study wave, as opposed to a diagnostic interview; however, WMH-CIDI data were available for a stratified subset of participants. Furthermore, one of these depression indicators asked whether a doctor had “ever told you that you have depression, stress or anxiety.” Although the diagnostic accuracy of this item in ROC analyses was reasonable (see Supplementary Table S2), it is possible that some respondents who scored positively experienced stress or anxiety rather than depression. However, it is also well known that depression and anxiety disorders are highly comorbid, and that there are typically high correlations between self-reported depression and anxiety measures [46]. On the other hand, while the two standardised symptom measures used here (K10 and PHQ-9) assessed a largely overlapping set of symptoms, feeling down/depressed and loss of interest/energy were amongst the strongest components (see Supplementary Table S4), which are the essential diagnostic features of depression. Moreover, in broad terms, the composite index appeared to work well, with lagged depression likelihood revealing linear associations with subsequent self-reported injury (Table 2) and maintenance of these exposure-outcome associations after adjusting for a range of other factors (Table 3). Furthermore, and following Rogan and Gladen’s approach [47], the “true prevalence” of lifetime affective disorder in the ARMHS baseline cohort was estimated to be 10.0% to 15.9% (see Supplementary Table S2), which was consistent with the national rate of 15.0% found in other Australian studies [48].

Thirdly, ARMHS also experienced sizeable attrition across the five years of data collection (see Supplementary Figure S2); consequently, although our data analysis techniques included all available data, it is possible that our findings are not representative of the target population. We also cannot exclude the possibility that effect estimates were influenced by participant loss to follow-up. Such bias could occur if loss was related to both depression (exposure) and injury (outcome) status. The presence or magnitude of such bias cannot be evaluated directly but we judge that it was unlikely to be substantial, since neither participant baseline depression nor injury status was significantly associated with the number of missing survey waves. In addition, several important factors mentioned in the introduction that may contribute to rural injury risk, such as occupation class, longer working hours, and greater risk-taking behaviours, were not measured in ARMHS and hence were not included in this analysis. Moreover, a direct comparison with metropolitan data was not possible here; however, such comparisons have been achieved for other aspects of psychosocial functioning by pooling ARMHS individual participant data with comparable longitudinal data from the Hunter Community Study [49].

## 5. Conclusions

Previous research has suggested a bi-directional relationship between depression and injury, with prior depression increasing the odds of subsequent unintentional injury; however, limited longitudinal research has explored these associations, particularly in rural contexts. In the current study, baseline cross-sectional analyses confirmed several previously observed associations with higher rates of 12-month unintentional injury, including being male, unemployed or unable to work, involved in a serious incident, and recent hazardous levels of alcohol use. In addition, there was a strong association between increasing depression likelihood and unintentional injury. The longitudinal analysis showed that probable depression was significantly associated with subsequent unintentional injury (OR 1.68), as was male gender (OR 1.39), while alcohol consumption was not found to mediate the relationship between depression likelihood and injury.

Given the observed relationships, it may be beneficial to consider unintentional injuries as a factor in public health campaigns to reduce mental illness and its consequences. Likewise, injury prevention initiatives may benefit from addressing mental health issues as a target. Such strategies may be particularly important in rural and remote areas where injuries are more common and mental health services are less readily available. It has also been proposed that injury prevention requires a contemporary approach, involving contributions from a range of sectors, including national, state and local governments, emergency services, non-government organisations, research institutes, and others, to adequately address this issue [50]. Hopefully, findings from studies such as the current one can add to the evidence base that underpins such injury prevention policies and initiatives.

## Figures and Tables

**Table 1:** Characteristics of Australian Rural Mental Health Study (ARMHS) participants at baseline: by likelihood of having experienced a recent depression episode, and by 12-month injury.

Characteristic	Likelihood of Recent Depression Episode: ^1^			12-Month Injury:	
	Unlikely (*N* = 1431) *N* (%)	Somewhat Likely (*N* = 579) *N* (%)	Probable (*N* = 611) *N* (%)	No (*N* = 2129) *N* (%)	Yes (*N* = 419) *N* (%)
**Age (years)—Mean (SD)**	56.9 (14.9)	53.4 (14.7)	52.5 (13.7) **	55.1 (14.7)	54.5 (14.4)
**Gender**					
Male	618 (43.2)	211 (36.4)	236 (38.6) *	829 (38.9)	208 (49.6) **
Female	813 (56.8)	368 (63.6)	375 (61.4)	1300 (61.1)	211 (50.4)
**Educational level**					
School Certificate (SC)/lower	410 (30.6)	165 (30.1)	167 (29.5)	611 (30.5)	110 (27.8)
Higher SC/trade/higher	930 (69.4)	383 (69.6)	400 (70.5)	1393 (69.5)	285 (72.2)
**Marital status**					
Married/de-facto	1139 (79.8)	423 (73.6)	401 (66.2) **	1617 (76.3)	302 (72.4)
Not partnered	289 (20.2)	152 (26.4)	205 (33.8)	503 (23.7)	115 (27.6)
**Employment status**					
Employed/studying/home-duties	862 (60.7)	380 (66.0)	368 (60.7) **	1318 (62.4)	265 (63.4) *
Unemployed/unable-to-work	57 (4.0)	32 (5.6)	114 (18.8)	144 (6.8)	47 (11.2)
Retired	501 (35.3)	164 (28.5)	124 (20.5)	649 (30.7)	106 (25.4)
**History of chronic disease**					
No	790 (55.2)	335 (57.9)	346 (56.6)	1206 (56.6)	227 (54.2)
Yes	641 (44.8)	244 (42.1)	265 (43.4)	923 (43.4)	192 (45.8)
**Serious accident**					
No	1389 (98.5)	553 (98.0)	573 (94.7) **	2072 (98.9)	381 (91.8) **
Yes	21 (1.5)	11 (2.0)	32 (5.3)	24 (1.1)	34 (8.2)
**Do you live on a farm**					
No	1047 (74.0)	441 (77.1)	454 (75.8)	1567 (74.7)	314 (75.7)
Yes	367 (26.0)	131 (22.9)	145 (24.2)	532 (25.3)	101 (24.3)
**ARIA+ 2006 ASGC category**					
Inner regional	544 (38.0)	217 (37.5)	249 (40.8)	831 (39.0)	152 (36.3)
Outer regional	508 (35.5)	204 (35.2)	216 (35.4)	755 (35.5)	154 (36.8)
Remote/very remote	379 (26.5)	158 (27.3)	146 (23.9)	542 (25.5)	113 (27.0)
**Alcohol consumption (AUDIT)**					
Low-risk	1111 (88.7)	421 (84.7)	432 (80.3) **	1620 (86.9)	288 (80.0) **
High-risk	142 (11.3)	76 (15.)	106 (19.7)	244 (13.1)	72 (20.0)
**Likelihood of recent depression episode**					
Unlikely				1216 (57.1)	182 (43.6) **
Somewhat likely				462 (21.7)	97 (23.3)
Probable				450 (21.1)	138 (33.1)

^1^ Eighteen participants were excluded from this analysis as they only completed one of the three recent depression episode indicators. Column percentages are reported together with overall tests of association with the baseline characteristic: * *p* < 0.01, ** *p* < 0.001.

**Table 2:** Frequencies of self-reported injury by lagged depression likelihood at Surveys 2–4.

Wave (Outcome)	Injury	Lagged Likelihood of Recent Depression Episode (i.e., Previous Wave):				Mantel-Haenszel Chi-Square ^1^	*p*-Value
		Unlikely *N* (%)	Somewhat Likely *N* (%)	Probable *N* (%)	Total *N* (%)		
Survey 2 (12 months)	No	799 (88.3)	332 (85.6)	230 (79.0)	1361 (85.9)		
	Yes	106 (11.7)	56 (14.4)	61 (21.0)	223 (14.1)	14.81	<0.001
Survey 3 (3 years)	No	648 (85.6)	231 (83.7)	169 (76.1)	1048 (83.5)		
	Yes	109 (14.4)	45 (16.3)	53 (23.9)	207 (16.5)	10.04	0.002
Survey 4 (5 years)	No	596 (88.2)	213 (84.2)	176 (83.4)	985 (86.4)		
	Yes	80 (11.8)	40 (15.8)	35 (16.6)	155 (13.6)	4.05	0.044

^1^ A test of the linear trend in injury frequencies across ordered depression categories.

**Table 3:** Univariate and multivariate logistic regression results, showing longitudinal associations of the lagged exposure and individual covariates with subsequent injury: Complete case analysis (*N* = 3669 exposure-outcome combinations, from 1754 unique participants).

Predictor (Lagged Exposure, Covariate)	Univariate Odds Ratio (99%CI)	Adjusted Odds Ratio (99%CI)
Likelihood of recent depression episode:		
Somewhat likely	1.23 (0.90, 1.67)	1.25 (0.90, 1.71)
Probable	1.72 (1.25, 2.35) **	1.68 (1.20, 2.35) **
Age (per 1 year increase)	1.00 (0.99, 1.01)	1.00 (0.99, 1.01)
Gender: Male	1.31 (1.00, 1.70) *	1.39 (1.06, 1.82) *
Education level: School Certificate/lower	1.03 (0.77, 1.37)	1.06 (0.77, 1.44)
Marital status: Not partnered	1.36 (1.01, 1.82) *	1.29 (0.95, 1.75) ^#^
Employment status:		
Unemployed/unable to work	1.18 (0.70, 1.98)	0.95 (0.55, 1.64)
Retired	1.00 (0.76, 1.31)	1.05 (0.72, 1.52)
History of chronic disease	0.90 (0.69, 1.17)	0.85 (0.63, 1.15)
Previous serious incident	1.51 (0.66, 3.46)	1.47 (0.65, 3.36)

Note: From GEE models accounting for clustering by household; see Table 1 for Reference categories associated with these Odds Ratios (e.g., Unlikely; Female gender; Married/de-facto): ^#^ *p* = 0.032, * *p* < 0.01, ** *p* < 0.001.

## Supplementary Materials

The following are available online at www.mdpi.com/1660-4601/14/9/1080/s1, Figure S1: Directed acyclic graph (DAG) showing the presumed causal relationship between the exposure, outcome and potential confounders/mediators, Figure S2: Patterns of participation across the four waves of the ARMHS project, Table S1: Characteristics of ARMHS participants: at baseline and by retention status, Table S2: Diagnostic accuracy analyses: used to identify/confirm cut-points for the three depression indicators and the composite index of the likelihood of a recent depression episode, Table S3: Cross-tabulated frequency distributions for the three depression indicators and the composite index of the likelihood of a recent depression episode by ARMHS survey wave, and Table S4: ARMHS symptom measures revisited (K10 and PHQ-9 item characteristics and profiles).
