# Supplementary material for: Depression and Risk of Unintentional Injury in Rural Communities—A Longitudinal Analysis of the Australian Rural Mental Health Study
Source: Int J Environ Res Public Health. 2017 Sep 18;14(9):1080. doi: 10.3390/ijerph14091080 (PMC5615617; doi:10.3390/ijerph14091080)
Supplement: Supplementary file 1 [file ijerph-14-01080-s001.pdf]

Supplementary Materials:

"*Depression and risk of unintentional injury in rural communities – a longitudinal analysis of the Australian Rural Mental Health Study*" (Inder at al.)

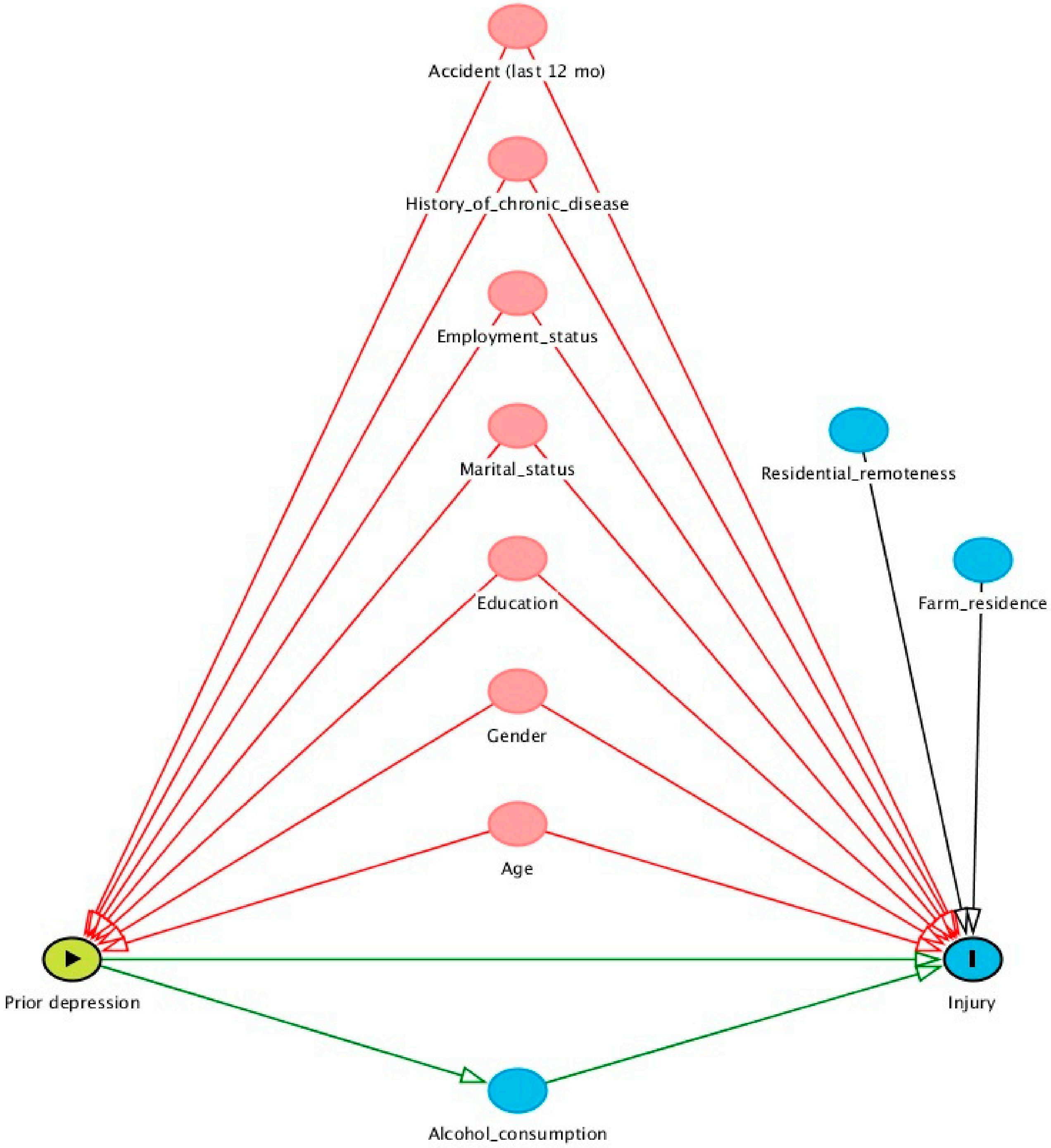

Figure S1. Directed acyclic graph (DAG) showing the presumed causal relationship between the exposure, outcome, and potential confounders/mediators.

Figure S2. Patterns of participation across the four waves of the ARMHS project.

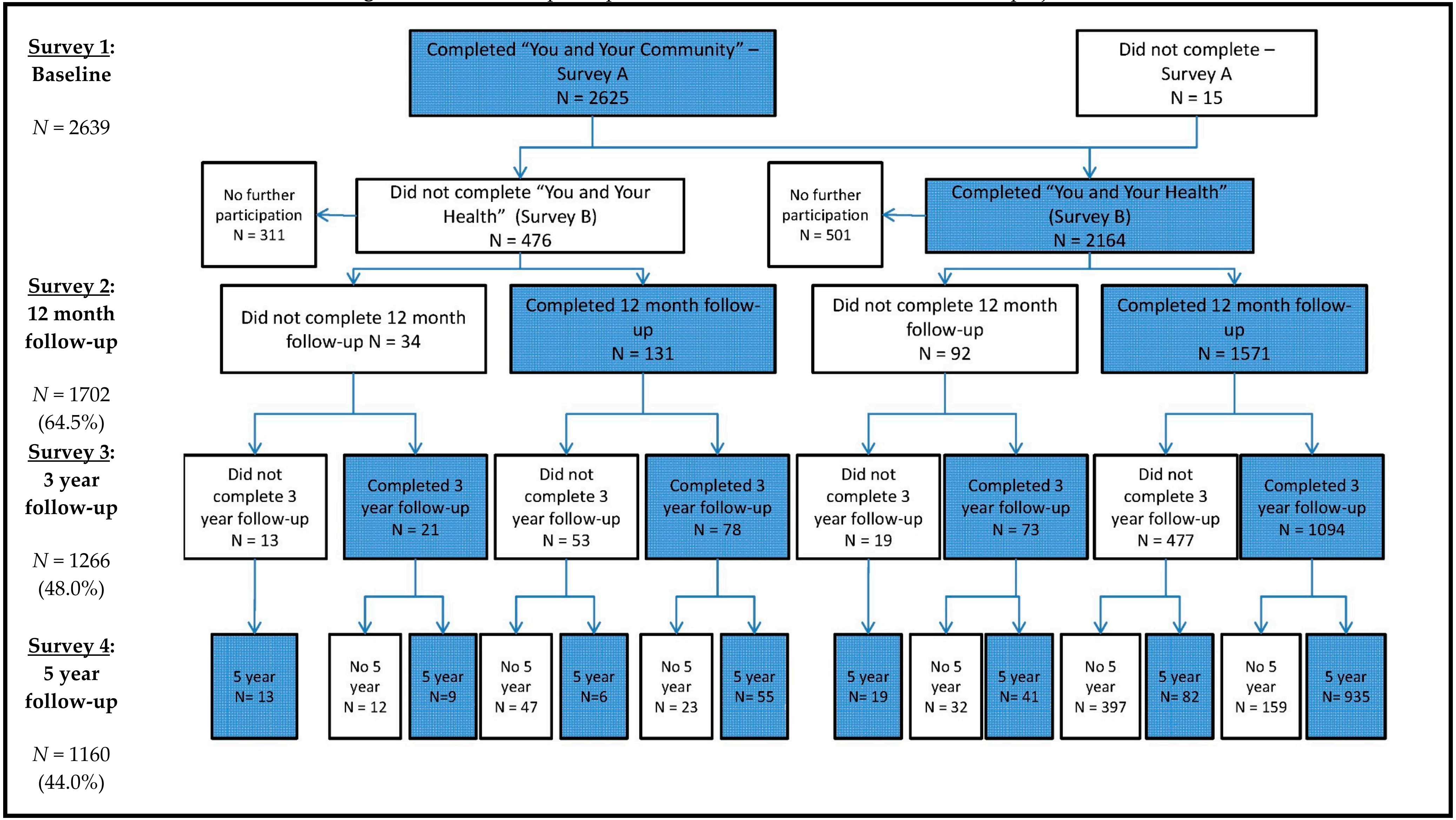

Table S1. Characteristics of ARMHS participants: at baseline and by retention status.

Characteristic	Overall baseline sample (N = 2621): [1] N (%)	At least one follow-up wave completed: No (N = 801) N (%)	Yes (N = 1820) N (%)
**Age (years) -** Mean (SD)	55.6 (14.7)	52.4 (16.0)	56.3 (13.9)**
**Gender**			
Male	1065 (40.6)	334 (41.7)	731 (40.2)
Female	1556 (59.4)	467 (58.3)	1089 (59.8)
**Educational level**			
School Certificate (SC)/lower	742 (30.2)	261 (35.4)	481 (28.0)**
Higher SC/trade/higher	1713 (69.8)	476 (64.6)	1237 (72.0)
**Marital status**			
Married/de-facto	1963 (75.2)	584 (73.4)	1379 (76.1)
Not partnered	646 (24.8)	212 (26.6)	434 (23.9)
**Employment status**			
Employed/studying/home-duties	1610 (61.9)	502 (63.5)	1108 (61.1)*
Unemployed/unable-to-work	203 (7.8)	76 (9.6)	127 (7.0)
Retired	789 (30.3)	212 (26.8)	577 (31.8)
**History of chronic disease**			
No	1471 (56.1)	474 (59.2)	997 (54.8)
Yes	1150 (43.9)	327 (40.8)	823 (45.2)
**Serious accident**			
No	2515 (97.5)	763 (97.2)	1752 (97.7)
Yes	64 (2.5)	22 (2.8)	42 (2.3)
**Do you live on a farm**			
No	1942 (75.1)	584 (74.0)	1358 (75.6)
Yes	643 (24.9)	205 (26.0)	438 (24.4)
**ARIA+ 2006 ASGC category**			
Inner regional	1010 (38.5)	296 (37.0)	714 (39.2)
Outer regional	928 (35.4)	269 (33.6)	659 (36.2)
Remote/very remote	683 (26.1)	236 (29.5)	447 (24.6)
**Alcohol consumption (AUDIT)**			
Low-risk	1964 (85.8)	422 (86.5)	1542 (85.7)
High-risk	324 (14.2)	66 (13.5)	258 (14.3)
**Likelihood of recent depression episode**			
Unlikely	1431 (54.6)	436 (54.4)	995 (54.7)
Somewhat likely	579 (22.1)	173 (21.6)	406 (22.3)
Probable	611 (23.3)	192 (24.0)	419 (23.0)

[1] Eighteen participants were excluded from this analysis as they only completed one of the three recent depression episode indicators. Column percentages are reported together with overall tests of association with retention status: * $p < 0.01$, ** $p < 0.001$.

Table S2. Diagnostic accuracy analyses: used to identify/confirm cut-points for the three depression indicators and the composite index of the likelihood of a recent depression episode.

**Baseline measure**	**ROC analyses of data from interviewed participants (stratified subset, N = 636), back-weighted to reflect baseline sample. Outcome: WHM-CIDI lifetime affective disorder diagnosis (Yes, *N* = 143; No, *N* = 493)**							
	**AUC**	**(99%CI)**	**Selected threshold (depression indicator)**	**% Above threshold**	**Sensitivity**	**Specificity**	**LR+**	**LR-**
i) Psychological distress symptoms (K10)	0.706	(0.644, 0.768)	> 15	31.5%	63.0%	73.5%	2.38	0.50
ii) Depression severity score (PHQ-9)	0.719	(0.652, 0.786)	≥ 5	20.6%	57.0%	85.3%	3.88	0.50
iii) Self-reported depression (Ever told by a doctor)	0.798	(0.736, 0.860)	Yes	28.8%	80.3%	79.4%	3.90	0.25
**Likelihood of recent depression episode** (Sum of 3 indicators above):								
“Probable” (≥ 2 indicators)	0.837	(0.788, 0.886)	≥ 2	25.0%	65.7%	81.4%	3.53	0.42
“Probable” or “Somewhat likely”			≥ 1	44.0%	94.7%	63.9%	2.62	0.08

Note: ROC, Receiver Operating Characteristic analysis; AUC, Area Under the Curve; LR+, Likelihood Ratio positive; LR-, Likelihood Ratio negative. Within the back-weighted data, the rates of lifetime affective disorder across the composite index categories were: “unlikely”, 1.4%; “somewhat likely”, 20.8%; and “probable”, 36.3%; with an overall rate of 13.8%. Following Rogan and Gladen’s approach [47], the ‘true prevalence’ of lifetime affective disorder was estimated by applying the tabled sensitivity and specificity values to the baseline distributions of recent depression likelihood from Table 1 (*N* = 2621): using the “probable” cut-point (611/2621), ‘true prevalence’ = 10.0% (99%CI: 5.5%, 14.5%); and using the “probable” or “somewhat likely” cut-point (1190/2621), ‘true prevalence’ = 15.9% (99%CI: 11.6%, 20.1%).

Table S3. Cross-tabulated frequency distributions for the three depression indicators and the composite index of the likelihood of a recent depression episode by ARMHS survey wave.

Likelihood of recent depression episode (during study wave)	Depression indicator (0/1) based on: K10	PHQ-9	Ever told by doctor	Survey 1 (Baseline, *N* = 2621) *N* (%)	Follow-up surveys: Survey 2 (12 months, *N* = 1675) *N* (%)	Survey 3 (3 years, *N* = 1249) *N* (%)	Survey 4 (5 years, *N* = 1151) *N* (%)	Overall (All waves, *N* = 6696) *N* (%)
Unlikely (0)	0	0	0	1431 (54.6)	967 (57.7)	722 (57.8)	641 (55.7)	3761 (56.2)
Somewhat likely (1)	1	0	0	251 (9.6)	92 (5.5)	53 (4.2)	15 (1.3)	411 (6.1)
	0	1	0	90 (3.4)	48 (2.9)	43 (3.4)	55 (4.8)	236 (3.5)
	0	0	1	238 (9.1)	206 (12.3)	190 (15.2)	197 (17.1)	831 (12.4)
Sub-total				*579 (22.1)*	*346 (20.7)*	*286 (22.9)*	*267 (23.2)*	*1478 (22.1)*
Probable (≥ 2)	1	1	0	164 (6.3)	112 (6.7)	57 (4.6)	52 (4.5)	385 (5.8)
	1	0	1	175 (6.7)	68 (4.1)	30 (2.4)	33 (2.9)	306 (4.6)
	0	1	1	45 (1.7)	34 (2.0)	22 (1.8)	49 (4.3)	150 (2.2)
	1	1	1	227 (8.7)	148 (8.8)	132 (10.6)	109 (9.5)	616 (9.2)
Sub-total				*611 (23.3)*	*362 (21.6)*	*241 (19.3)*	*243 (21.1)*	*1457 (21.8)*

Note: Likelihood of recent depression episode (during study wave) was based on three (0/1) depression indicators: K10 > 15 (for wave); PHQ-9 ≥ 5 (for wave); and ever being told by a doctor that you have '*depression, stress or anxiety*' (cumulative across waves). Survey data were excluded from this analysis if only one of these three recent depression indicators was available. Across the study waves, 1457 surveys were categorized as indicating a recent 'probable depression' episode, amongst which 42.3% (616) were positive on all three indicators, 31.3% (456) were positive on the 'ever told by doctor' indicator plus one other, and a further 26.4% (385) were positive on both symptom measures (K10, PHQ-9).

Table S4. ARMHS symptom measures revisited (K10 and PHQ-9 item characteristics and profiles).

Table S4A. K10 and PHQ-9 item characteristics (internal consistency, cross-correlations, component loadings): N = 2626 individuals, 4 ARMHS waves (6659 sets of K10 ratings; 6318 sets of PHQ-9 ratings)

**Measure – Item**	**Corrected item - scale correlation** (K10 or PHQ-9 total minus item)	**Cross-correlation with other scale** (K10 or PHQ-9 total)	**Loading on First Principal Component** (All 19 items)
**Kessler Psychological Distress Scale (K10) – last 4 weeks:**			
Q7: Depressed	0.773	0.676	0.811
Q4: Hopeless	0.733	0.620	0.775
Q10: Worthless	0.714	0.625	0.774
Q8: That everything was an effort	0.726	0.658	0.764
Q9: So sad that nothing could cheer you up	0.723	0.599	0.763
Q5: Restless or fidgety	0.648	0.521	0.664
Q2: Nervous	0.618	0.500	0.639
Q6: So restless you could not sit still	0.595	0.464	0.619
Q1: Tired out for no good reason	0.651	0.543	0.613
Q3: So nervous that nothing could calm you down	0.591	0.439	0.605
**Patient-Health Questionnaire-9 (PHQ-9) - last 2 weeks:**			
Q2: Feeling down, depressed or hopeless	0.743	0.695	0.803
Q1: Little interest or pleasure in doing things	0.721	0.646	0.759
Q6: Feeling bad about yourself, or that you are a failure, or have let yourself or your family down	0.680	0.640	0.751
Q7: Trouble concentrating on things, such as reading the newspaper or watching television	0.655	0.564	0.684
Q4: Feeling tired or little energy	0.653	0.573	0.655
Q5: Poor appetite or overeating	0.632	0.533	0.646
Q9: Thoughts that you would be better off dead, or of hurting yourself in some way	0.539	0.500	0.615
Q3: Trouble falling or staying asleep, or sleeping too much	0.597	0.498	0.598
Q8: Moving or speaking so slowly that other people could have noticed. Or the opposite: being so fidgety or restless that you have been moving around a lot more than usual	0.549	0.467	0.581

Note: K10 items were rated on 1 ('*None of the time*') to 5 ('*All of the time*') scales, while PHQ-9 items were rated on 0 ('*Not at all*') to 3 ('*Nearly every day*') scales. Internal consistency (Cronbach alpha) coefficients: K10, 0.898; PHQ-9, 0.878; all 19 items, 0.932. Items in this table are ranked (within scales) by their loading on the first principal component (from an analysis including all 19 items). The first principal component accounted for 48.26% of the item variance (eigenvalue = 9.169), with all items having moderate to high loadings. Correlation between K10 and PHQ-9 total scores, 0.780 (N = 6262 sets of ratings). Using the cut-points for the binary depression indicators [K10 > 15 (for wave); PHQ-9 ≥ 5 (for wave)], 24.9% of K10 scores were positive and 22.0% of PHQ-9 scores, with 85.0% concordance (overall Kappa coefficient, 0.584): 1001 (16.0%) above threshold on both; 560 (8.9%) only on the K10; 378 (6.0%) only on the PHQ-9; and 4323 (69.0%) below threshold on both.

Table S4B. K10 and PHQ-9 item profiles (mean ratings) across depression likelihood index scores/categories: *N* = 2626 individuals, 4 ARMHS waves (6659 sets of K10 ratings; 6318 sets of PHQ-9 ratings).

**Measure – Item** (Mean ratings)	**Likelihood of recent depression episode (during study wave)**				**Wald chi-squares for polynomial contrasts from GEE analyses** (Trend components of change across depression likelihood)		
	(Unlikely)	(Somewhat likely)	(Probable)				
	**0**	**1**	**2**	**3**	**Linear**	**Quadratic**	**Cubic**
**Kessler Psychological Distress Scale (K10) – last 4 weeks:**							
K10 total score	11.36	13.95	18.95	23.33	1,385.31**	18.65**	16.86**
Q7: Depressed	1.13	1.51	2.20	2.87	1,414.69**	22.15**	8.32*
Q8: That everything was an effort	1.22	1.58	2.35	2.91	1,290.17**	8.86*	24.02**
Q1: Tired out for no good reason	1.57	2.02	2.71	3.14	1,134.69**	0.12	13.09**
Q2: Nervous	1.26	1.65	2.16	2.51	805.90**	0.84	5.06
Q5: Restless or fidgety	1.15	1.47	1.96	2.31	658.98*	0.33	7.25*
Q4: Hopeless	1.02	1.19	1.69	2.14	588.56**	20.87**	9.74*
Q9: So sad that nothing could cheer you up	1.01	1.16	1.58	2.07	510.86**	33.95**	4.04
Q10: Worthless	1.00	1.15	1.58	2.12	398.59**	29.29**	1.79
Q6: So restless you could not sit still	1.01	1.15	1.44	1.72	252.20**	7.18*	2.61
Q3: So nervous that nothing could calm you down	1.00	1.08	1.28	1.54	214.37**	14.84**	0.95
**Patient-Health Questionnaire-9 (PHQ-9) - last 2 weeks:**							
PHQ-9 total score	1.07	2.67	6.33	10.22	1,456.15**	53.38**	10.75*
Q2: Feeling down, depressed or hopeless	0.06	0.26	0.74	1.30	1,251.31**	54.58**	4.86
Q4: Feeling tired or little energy	0.42	0.77	1.37	1.72	1,173.61**	0.00	17.95**
Q1: Little interest or pleasure in doing things	0.07	0.24	0.75	1.25	1,070.99**	40.24**	12.55**
Q3: Trouble falling or staying asleep, or sleeping too much	0.34	0.70	1.26	1.61	927.79**	0.05	8.14*
Q5: Poor appetite or overeating	0.11	0.32	0.79	1.22	670.06**	12.61**	5.66
Q6: Feeling bad about yourself, or that you are a failure, or have let yourself or your family down	0.03	0.18	0.58	1.10	594.45**	43.53**	1.76
Q7: Trouble concentrating on things, such as reading the newspaper or watching television	0.05	0.17	0.54	1.04	537.44**	47.40**	1.38
Q8: Moving or speaking so slowly that other people could have noticed. Or the opposite: being so fidgety or restless that you have been moving around a lot more than usual	0.01	0.06	0.21	0.57	236.66**	47.66**	2.61
Q9: Thoughts that you would be better off dead, or of hurting yourself in some way	0.00	0.02	0.15	0.43	138.68**	36.06**	0.86

Note: Likelihood of recent depression episode (during study wave) was based on three (0/1) depression indicators: K10 > 15 (for wave); PHQ-9 ≥ 5 (for wave); and ever being told by a doctor that you have '*depression, stress or anxiety*' (cumulative across waves). A separate generalized estimating equation (GEE) analysis was conducted for each item, controlling for study wave and within individual variation. Items in this table are ranked (within scales) by the overall strength of association with the depression likelihood index. Strong linear components of trend were evident for all 19 items, modest quadratic components for 14 items, and small cubic components for 8 items (with the non-linear elements being largely influenced by less marked differences between the 0 and 1 scores on the depression likelihood index): * $p < 0.01$; ** $p < 0.001$.
